# Electrical Stimulation and Motor Function Rehabilitation in Spinal Cord Injury: A Systematic Review

**DOI:** 10.7759/cureus.61436

**Published:** 2024-05-31

**Authors:** Asterios K Kanakis, Ioannis S Benetos, Dimitrios Stergios Evangelopoulos, John Vlamis, Elias S Vasiliadis, Aikaterini Kotroni, Spyros G Pneumaticos

**Affiliations:** 1 Department of Physical Medicine and Rehabilitation, KAT Hospital, Athens, GRC; 2 3rd Department of Orthopaedic Surgery, National and Kapodistrian University of Athens (NKUA) KAT Hospital, Athens, GRC

**Keywords:** motor recovery, function, functional electrical stimulation (fes), transcutaneous spinal cord stimulation (tscs), epidural spinal cord stimulation (escs), electrical stimulation, spinal cord stimulation, spinal cord injury

## Abstract

Spinal cord injury (SCI) often leads to devastating motor impairments, significantly affecting the quality of life of affected individuals. Over the last decades, spinal cord electrical stimulation seems to have encouraging effects on the motor recovery of impacted patients. This review aimed to identify clinical trials focused on motor function recovery through the application of epidural electrical stimulation, transcutaneous electrical stimulation, and functional electrical stimulation. Several clinical trials met these criteria, focusing on the impact of the aforementioned interventions on walking, standing, swimming, trunk stability, and upper extremity functionality, particularly grasp. After a thorough PubMed online database research, 37 clinical trials were included in this review, with a total of 192 patients. Many of them appeared to have an improvement in function, either clinically assessed or recorded through electromyography. This review outlines the various ways electrical stimulation techniques can aid in the motor recovery of SCI patients. It stresses the ongoing need for medical research to refine these techniques and ultimately enhance rehabilitation results in clinical settings.

## Introduction and background

Annually, an estimated half million new cases of spinal cord injury (SCI) occur globally, with the majority of these incidents stemming from preventable causes such as vehicular accidents (38%), falls (30%), violence (13%), sports injuries (9%), and medical and surgical etiologies (5%) [1].

SCI frequently results in profound motor deficits, severely compromising the well-being and daily functioning of those impacted. SCI commonly arises as a consequence of trauma, resulting in the loss of sensory, motor, and/or autonomic functions [2]. The primary injury, resulting from the initial mechanical trauma to the spinal cord, involves damage to neural parenchyma, disruption of axonal networks, and disturbance of glial membranes [3]. After the initial insult, further harm to the injured spinal cord can occur through various mechanisms such as apoptotic signaling, ischemia, excitotoxicity, inflammation, and axonal demyelination. As a result of these local events, known as secondary injury, glial scar formation occurs. This formation presents a significant challenge, as it can hinder axonal regeneration and synaptic neuroplasticity at the injury site, thereby exacerbating the recovery process for individuals with SCIs [2]. With the aforementioned factors, a disruption occurs within the spinal cord, halting the transmission of signals from the upper motor neurons to areas below the level of injury. However, in most injuries, it seems that propriospinal connections can often offer an indirect pathway for accessing afferent signals [4]. Recent studies suggest that leveraging existing neural connections can facilitate functional recovery by restoring sensorimotor function [5]. Contemporary clinical protocols prioritize prompt surgical decompression and mechanical stabilization of the SCI site. Subsequent pharmacological interventions, including medications such as methylprednisolone, nimodipine, naloxone, and others, are commonly administered to mitigate further damage and facilitate recovery [6,7]. A rehabilitation program typically follows, with the aim of fostering patient independence by retraining them in activities of daily living (ADLs) and restoring functionality based on the nature of the injury. Unfortunately, none of those approaches appear to affect neuroregeneration and functional recovery. Spinal cord stimulation (SCS), whether epidural or transcutaneous, is a technique that has been studied and appears to take advantage of the existing intact neurological connections within the spinal cord, promoting functional recovery. These remaining fibers play a crucial role in facilitating plasticity by establishing communication pathways across the spinal cord lesion.

Epidural electrical stimulation involves the direct application of electrical currents to the spinal cord, targeting specific motor circuits to facilitate voluntary movement. Transcutaneous electrical stimulation utilizes surface electrodes to deliver electrical impulses, stimulating peripheral nerves and potentially enhancing motor function. However, none of these techniques have been clinically applied so far. On the other hand, functional electrical stimulation (FES) is a procedure that has already been used through the years for neurorehabilitation. It can be applied to various locations, including nerves, muscles, and skin, and is often combined with specific tasks as part of the rehabilitation program. FES involves the synchronized activation of muscles to produce functional movements, often through the use of external devices. Findings suggest that epidural electrical stimulation holds promise in eliciting significant improvements in motor function, particularly in individuals with severe SCI. Transcutaneous electrical stimulation demonstrates potential benefits, particularly in pain management and muscle activation. FES shows promise in promoting task-specific motor training and facilitating functional movement patterns.

## Review

Study design

This review aimed to focus on clinical trials related to motor recovery after SCI, specifically when electrical stimulation techniques such as epidural and transcutaneous SCS and FES were utilized.

A literature search was performed according to the Preferred Reporting Items for Systematic Reviews and Meta-Analyses (PRISMA) guidelines [8]. PubMed was the selected database for this purpose, and the following keywords were applied: spinal cord injury, spinal cord stimulation, epidural stimulation, transcutaneous stimulation, electrical stimulation, functional electrical stimulation, motor recovery, function, combined as follows: (“spinal cord injury” OR SCI) AND (“electrical stimulation” OR “spinal cord stimulation” OR “functional electrical stimulation” OR FES OR “epidural stimulation” OR EES OR “transcutaneous spinal cord stimulation” OR tSCS) AND (motor OR locomotor OR funtion* OR walk* OR “motor recovery” OR “motor response” OR recovery OR rehabilitation OR gait).

The following inclusion criteria were applied in this review: clinical trials involving human patients with SCIs, the application of electrical stimulation, and outcomes specifically related to motor recovery. An additional filter, limited to the past 10 years, was exclusively applied for FES, and only articles meeting this criterion were included in the review.

**Figure 1:**
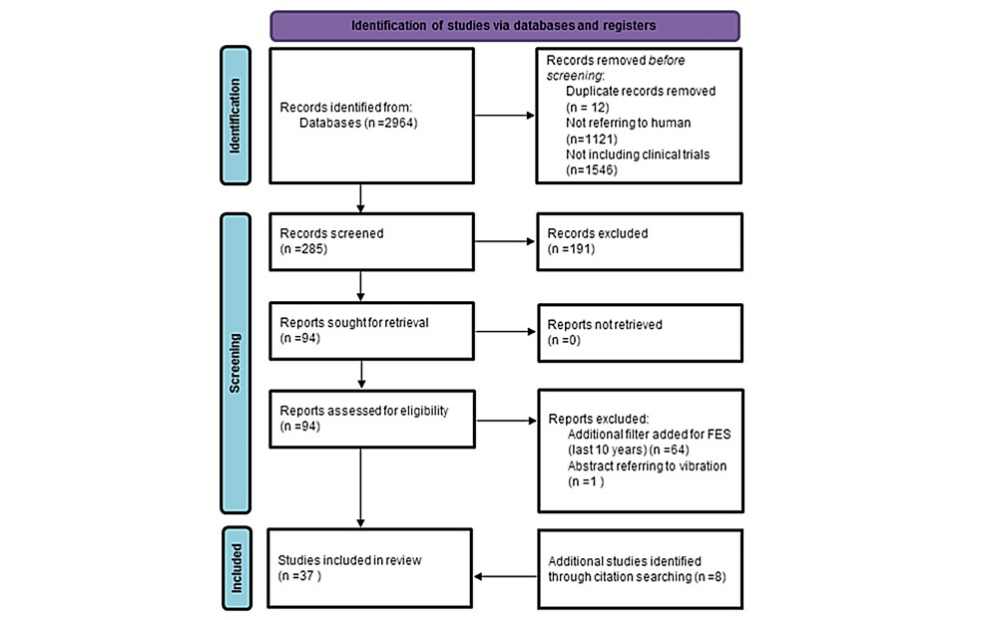
Flow diagram. FES = functional electrical stimulation

A total of 2,964 articles were identified using the pre-specified keywords. Of them, 1,843 articles were related to humans, and 297 were identified as clinical trials. Following title screening, 191 abstracts were excluded due to their lack of relevance to motor recovery. Additionally, duplicate articles were removed. After applying the additional filter for FES, 37 articles were selected for full-text reading. The study flow diagram presents the selection process (Figure 1).

Epidural spinal cord stimulation

Epidural spinal cord stimulation (eSCS) is an advanced therapeutic approach specifically designed to address motor dysfunction in individuals with SCI. This technique involves the precise placement of electrodes within the epidural space of the spinal cord, strategically positioned to target regions associated with motor control and coordination. By delivering controlled electrical impulses directly to these neural pathways, eSCS aims to modulate the activity of motor circuits, promote neuroplasticity, and facilitate functional recovery below the level of injury [9]. The mechanism of action of eSCS in motor recovery centers on its ability to modulate neural activity within the injured spinal cord. Through the application of electrical stimulation, eSCS can bypass damaged areas of the spinal cord and directly activate intact neural circuits responsible for generating motor commands. By artificially inducing neuronal firing patterns, eSCS may help re-establish communication pathways between the brain and the muscles, enabling individuals with SCI to regain voluntary control and coordination of movement [10]. Furthermore, eSCS has been shown to promote neuroplastic changes within the spinal cord, facilitating the formation of new synaptic connections and strengthening existing neural pathways. This process, known as neural reorganization, plays a crucial role in motor recovery by enhancing the adaptability and flexibility of the spinal cord circuitry. By promoting adaptive changes in response to electrical stimulation, eSCS can help optimize motor function and improve muscle activation patterns in individuals with SCI [10]. One of the key advantages of eSCS in motor recovery is its ability to provide targeted and adjustable stimulation parameters. Healthcare providers can customize the frequency, amplitude, and pulse width of the electrical impulses to suit the specific needs and capabilities of each patient. This personalized approach allows for precise modulation of neural activity, maximizing the therapeutic benefits while minimizing potential side effects. Overall, eSCS shows promise as an intervention for promoting motor recovery in individuals with SCI. By harnessing the power of electrical stimulation to modulate neural circuits and promote neuroplasticity, eSCS offers a novel approach to enhancing motor function and improving the quality of life of individuals living with SCI. Ongoing research endeavors continue to explore the full potential of eSCS in motor rehabilitation to optimize outcomes and expand its utility across a broader range of neurological conditions.

In a 2023 study by Gorgey et al., findings revealed promising results for two individuals with chronic SCI, particularly in terms of standing ability [11]. Another clinical trial involving two subjects, conducted by Darrow et al., emphasized electromyography (EMG) responses and demonstrated enhanced muscle strength following five visits [12]. In a 2019 clinical trial by Calvert et al., motor function was detected in two individuals with SCI, within the first five days of eSCS [13]. Additionally, Angeli et al. (2014) described volitional leg movements in four patients [14]. Furthermore, supraspinal control of some leg movements and locomotor patterns was recorded only during stimulation in a 2011 clinical trial by Harkema et al. [15]. In a 2017 follow-up of the previous subject, Rejc et al. described the outcomes of volitional lower limb movement and standing recovery without stimulation [16]. In a 2017 case report by Grahn et al., volitional control (including task-specific muscle activity, step-like movements, and full weight-bearing independent standing) was described in a patient after eight sessions of stimulation [17]. A follow-up conducted 43 weeks later by Gill et al. demonstrated further improvements, including bilateral stepping on a treadmill, independent body weight support, and independent overground stepping [18]. EMG findings were recorded during the extension of the lower limbs in five participants in a 2002 clinical trial by Jilge et al. [19]. In a study by Lu et al. in 2016, an increase in grip strength and control was observed in two subjects. This improvement was further supported by an increase in the International Standards for Neurological Classification of Spinal Cord Injury upper extremity motor score [20]. Likewise, step-like movements and EMG activity in flexors and extensors appeared in a 10-participant clinical trial by Minassian et al. in 2004 [21]. Additionally, improvements in double walking speed and a reduction in effort for overground walking were suggested in a 2004 study by Carhart et al. [22]. Likewise, improved walking speed, endurance, and a reduced sense of effort were observed in a 2006 clinical trial involving two subjects conducted by Huang et al. [23]. Finally, in a study by Dy et al. in 2010, involving 10 patients with chronic SCI, significant phase-dependent modulation was observed. This finding suggests compelling evidence that the human lumbosacral spinal circuitry can regulate afferent input based on the phase of the step cycle even without input from the brain [24].

Table 1 displays the eSCS studies incorporated in this analysis, presenting the demographic characteristics, injury type/level, stimulator type and location, and the primary outcomes of each study.

**Table 1:** Overview of epidural spinal cord stimulation studies. AIS = American Spinal Injury Association Impairment Scale; NS = not specified; M = male; F = female; C = cervical; T = thoracic; L: lumbar; S = sacral; EMG = electromyography; ISNCSCI = International Standards for Neurological Classification of Spinal Cord Injury

Study	Design	Subjects	Sex	Time	Intervention	Level - AIS	Stimulator type	Location of stimuli	Outcome
Gorgey et al. (2023) [11]	Case study	2	NS	Chronic	Percutaneous	C8 AIS A, T11 AIS B	Medtronic	T10-L2	Voluntary motor activity in one subject (T11), standing ability in both subjects, exoskeleton/assisted walking/overground stepping: only EMG (±) for the C8 subject and positive for the T11 subject
Darrow et al. (2019) [12]	Clinical trial	2	F	Chronic	Paddle	T8 AIS A, T4 AIS A	Abbott	L1-S2	EMG response: in both subjects, volitional activity. In both (but not functional for the T4 subject), increased muscle strength after five visits: in both subjects
Calvert et al. (2019) [13]	Clinical trial	2	M	Chronic	Paddle	T6 AIS A, T3 AIS A	Medtronic	T11-L1	Motor function within the first five days: in both subjects
Angeli et al. (2014) [14]	Clinical trial	4	M	Chronic	Paddle	T2 AIS B, T4 AIS A, C7 AIS B, T5 AIS A	Medtronic	T11-T12	Volitional leg movements: detected in all subjects
Harkema et al. (2011) [15]	Case report	1	M	Chronic	Paddle	C7 AIS B	Medtronic	L1-S1	Evidence of supraspinal control of some leg movements, locomotor-like patterns
Rejc et al. (2017) [16]. Follow-up of [15] after 3.7 years	Clinical trial	1	M	Chronic	Paddle	C7 AIS B	Medtronic	T11-L1	Volitional lower limb movement and standing recovery without stimulation
Grahn et al. (2017) [17]	Case report	1	M	Chronic	Paddle	T6 AIS A	Medtronic	NS	After eight sessions: volitional control reported (task-specific muscle activity, step-like movements, full weight-bearing independent standing)
Gill et al. (2018) [18]. Follow-up of [17]	Case report	1	M	Chronic	Paddle	T6 AIS A	Medtronic	T11-L1	43 weeks later: bilateral stepping on a treadmill, independent body weight support, independent stepping overground
Jilge et al. (2002) [19]	Clinical trial	5	3 F, 2 M	Chronic	Percutaneous	4 AIS A, 1 AIS B	Medtronic	T12-L1	Extension of the lower limbs: all subjects provided EMG findings
Lu et al. (2016) [20]	Case report	2	M	Chronic	Paddle	C5 AIS B, C6 AIS B	Boston Scientific	C5-T1	Grip strength/control increased, ISNCSCI upper extremity motor score improved from 9 to 32 for the first subject and from 17 to 23 for the other
Minassian et al. (2004) [21]	Clinical trial	10	7 M, 3 F	Chronic	Percutaneous	C6 AIS A, C4 AIS A, C4 AIS A, C7 AIS B, T10 AIS B, T4 AIS A, T6 AIS A, T4 AIS A, T5 AIS A, T7 AIS A	Medtronic	T10 to L1	Stepping-like movements and EMG activity in flexors and extensors detected in all subjects
Carhart et al. (2004) [22]	Case report	1	NS	Chronic	Percutaneous	C5 AIS C	Medtronic	T10-T12	Improvement in treadmill, over-ground ambulation, reduction in an effort for over-ground walking, double-walking speed
Huang et al. (2006) [23]	Clinical trial	2	M	Chronic	Percutaneous	C5 AIS C, T8 AIS C	Medtronic	T10-L2	Improved walking speed, endurance, and reduced sense of effort, in both subjects
Dy et al. (2010) [24]	Clinical trial	9	M	Chronic	Percutaneous	C5-T7 AIS A	Lead-Lok, Sandpoint, ID	T11-T12	Significant phase-dependent modulation was observed in all subjects, evidence that in the absence of input from the brain, the human lumbosacral spinal circuitry can gate afferent input as a function of the phase of the step cycle

Transcutaneous spinal cord stimulation

Transcutaneous spinal cord stimulation (tSCS) is an innovative therapeutic approach to modulate spinal cord excitability through non-invasive electrical stimulation applied to the skin overlying the spinal cord. This technique has gained increasing attention in recent years due to its potential to promote neural plasticity and enhance motor function in individuals with SCI [25]. tSCS involves the application of electrical currents to the skin surface, typically using adhesive electrodes placed along the spinal column at specific dermatomal levels corresponding to the desired target region. These electrodes deliver electrical impulses that penetrate the skin and underlying tissues, reaching the spinal cord to modulate neuronal activity [26]. The mechanisms underlying the therapeutic effects of tSCS are multifaceted and not yet fully understood. However, it is believed that tSCS may exert its effects by directly influencing the excitability of spinal cord circuits, as well as by activating descending and ascending pathways involved in motor control and sensory processing [27,28]. By delivering controlled electrical stimulation to the spinal cord, tSCS can modulate neuronal firing patterns, enhance synaptic transmission, and promote the release of neurotransmitters implicated in motor function and sensory perception [5,29]. One of the key advantages of tSCS is its non-invasive nature, which eliminates the need for surgical implantation of electrodes into the spinal canal. Instead, tSCS electrodes are placed on the skin surface, allowing for easy application and removal without the risks associated with invasive procedures. This makes tSCS a safer and more accessible option for individuals with SCI who may not be suitable candidates for surgical interventions or who prefer non-invasive treatment modalities [30]. In clinical practice, tSCS is typically administered using portable stimulator devices that deliver adjustable electrical currents to the skin electrodes. These stimulators allow for precise control over stimulation parameters, including pulse frequency, intensity, and duration, enabling tailored treatment protocols to meet individual patient needs. tSCS sessions are often conducted in outpatient settings under the supervision of trained healthcare professionals, who monitor patient response and adjust stimulation parameters as necessary to optimize therapeutic outcomes [30]. The therapeutic efficacy of tSCS has been investigated in numerous clinical studies evaluating its effects on motor function, sensory perception, and other neurological outcomes in individuals with SCI [31]. Research findings suggest that tSCS may have beneficial effects on muscle strength, voluntary movement, spasticity, and neuropathic pain in some patients [32]. However, the optimal stimulation parameters and treatment protocols for maximizing the therapeutic benefits of tSCS remain areas of ongoing investigation. Overall, tSCS represents a promising therapeutic modality for enhancing motor function and promoting recovery in individuals with SCI. Its non-invasive nature, adjustable stimulation parameters, and potential to modulate spinal cord excitability make it a valuable addition to the armamentarium of rehabilitative interventions for SCI. Continued research efforts are needed to further elucidate the underlying mechanisms of tSCS and optimize its clinical implementation for maximizing patient outcomes.

In a randomized control trial including 10 participants with cervical SCI, Huang et al. observed EMG volitional muscle activation in all subjects, while half of them generated a grip force, when tSCS combined with buspirone was applied [33]. Furthermore, Gad et al. described the achievement of voluntary hand grip in just one session, along with an increase in maximum grip strength, in six individuals [34]. Evidence of neuromodulating the spinal locomotor circuit was provided by Gerasimenko et al., who observed induced rhythmic leg movements and corresponding coordinated movement EMG activity in leg muscles with stimulation after studying five subjects [30]. Rath et al., in an eight-participant clinical trial, yielded positive outcomes regarding trunk stability, indicating a more stable and upright sitting posture [35]. In a clinical trial by Hofstoetter et al., increased hip flexion during swing was observed in three individuals while tSCS was applied [36]. In addition, outcomes from a clinical trial involving three subjects, conducted by Minassian et al., revealed rhythmic motor outputs in some lower limb muscles [37]. Similarly, a case report by Hofstoetter et al. observed enhanced EMG activities during a step-phase and coordination of hip and knee movements, indicative of voluntary locomotor control [32]. Moreover, all subjects in a clinical trial of six participants by Inanici et al. showed rapid signs of recovery in hand and arm function [38]. Sayenko et al. presented findings on maintaining upright standing. In a clinical trial involving 15 individuals, eight required minimal assistance, while seven required no assistance to maintain that standing [39]. In another clinical trial conducted by Inanici et al., which involved one subject with incomplete cervical SCI, an improvement in the neurological level of injury was observed, progressing from C3 to C4 [40]. In a study by Freyvert et.al., improved hand strength and voluntary control were described in a clinical trial with six participants, when tSCS was combined with buspirone [41]. Finally, an enhanced level of effort while stepping was reported in a case report by Gad et al., where tSCS, exoskeleton, and buspirone were combined [42].

Table 2 summarizes the tSCS studies incorporated in this analysis, presenting the demographic characteristics, injury type/level, stimulator type and location, and the primary outcomes of each study.

**Table 2:** Overview of transcutaneous spinal cord stimulation studies. tSCS = transcutaneous spinal cord stimulation; AIS = American Spinal Injury Association Impairment Scale; NS = not specified; M = male; F = female; C = cervical; T = thoracic; L = lumbar; S = sacral; Co = coccyx; EMG = electromyography; NLI = neurological level of injury; PT = physical therapy

Study	Design	Subjects	Sex	Time	Interventions	Level - AIS	Stimulator type	Location of stimuli	Outcome
Huang et al. (2022) [33]	Randomized control trial	10	6 M, 4 F	Chronic	tSCS + buspirone	C3-C7 (5) AIS A, (5) AIS B	NeuroRecovery Technologies Inc	C4-C5	EMG volitional muscle activation in all subjects generated a grip force in half of the subjects
Gad et al. (2018) [34]	Clinical trial	6	5 M, 1 F	Chronic	tSCS	C4 AIS C, C6 AIS C, C6 AIS C, C4 AIS B, C8 AIS C, C4 AIS B	NeuroRecovery Technologies Inc	C3-C4/C6-C7	Voluntary hand grip and maximum grip strength increased in all subjects
Gerasimenko et al. (2015) [30]	Clinical trial	5	M	Chronic	tSCS	C5-T3 AIS NS	Axelgaard, Fallbrook, CA	T11/over Coccyx 1	Evidence of neuromodulating the spinal locomotor circuit in all subjects
Rath et al. (2018) [35]	Clinical trial	8	7 M, 1 F	Chronic	tSCS	T4 AIS A,T2 AIS A,T9 AIS A, T2 AIS A, C4 AIS C, T3 AIS A, C5 AIS C, T3 AIS A	ValuTrode, Axelgaard Ltd., Fallbrook, CA	T11-T12/L1-L2	Improved trunk stability, more stable, erect sitting posture in all subjects
Hofstoetter et al. (2015) [36]	Clinical trial	3	2 M, 1 F	Chronic	tSCS	T9 AIS D, C5 AIS D, C5 AIS D	Schwa-medico GmbH, Ehringshausen, Germany	T11-T12	Increased hip flexion during swing in all subjects
Minassian et al. (2016) [37]	Clinical trial	4	3 M, 1 F	Chronic	tSCS + robotic-driven gait orthosis	C8-T8 AIS A	Schwa-Medico	T11-T12	Rhythmic motor outputs were produced in some lower limb muscles of each subject
Hofstoetter et al. (2013) [32]	Case report	1	F	Chronic	tSCS + treadmill stepping	T9 AIS D	Schwa-Medico	T11-T12	Voluntary locomotor control was reported
Inanici et al. (2021) [38]	Clinical trial	6	4 M, 2 F	Chronic	tSCS + functional task training	C5 AIS B, C5 AIS B, C5 AIS C,C5 AIS C, C5 AIS C, C3 AIS D	NeuroRecovery Technologies Inc.	C2+C4 or C4+C6, anterior iliac crests of pelvis	Recovery of hand and arm function in all subjects
Sayenko et al. (2019) [39]	Clinical trial	15	12 M, 3 F	Chronic	tSCS + locomotor training	C4–T12 (11)AIS A, (1) AIS B, (3) AIS C	ValuTrode	T11-T12, L1-L2	All maintained upright standing with minimum (eight subjects) and without (seven subjects) external assistance
Inanici et al. (2018) [40]	Clinical trial	1	M	Chronic	tSCS + PT	C3 AIS D	NeuroRecovery Technologies Inc.	C3-C4, C6-C7	NLI improved from C3 to C4
Freyvert et al. (2018) [41]	Clinical trial	6	4 M, 2 F	Chronic	tSCS + buspirone	C2-C6 AIS B	NeuroRecovery Technologies Inc.	C5	Improved hand strength and voluntary control in all subjects
Gad et al. (2017) [42]	Case report	1	M	Chronic	tSCS + exoskeleton + buspirone	T9 AIS A	Axelgaard, Fallbrook, CA	T11-T12, Co1	Enhanced the level of effort while stepping

Functional electrical stimulation

FES is a therapeutic approach that holds promise for individuals with SCI by aiming to restore lost motor function. This technique involves the use of electrical currents to stimulate nerves and muscles, eliciting controlled muscle contractions and movements [43,44]. In the context of SCI, FES is particularly beneficial for individuals with partial or complete paralysis, as it bypasses the damaged neural pathways to directly activate muscles [43]. By delivering electrical impulses to the muscles or nerves, FES can evoke functional movements such as grasping, walking, or standing, thus promoting motor recovery. The application of FES in SCI rehabilitation typically involves the use of specialized devices such as FES bikes, foot drop stimulators, or hand grasp systems [45]. These devices are designed to deliver precise electrical stimulation to specific muscle groups or nerves corresponding to the desired movement. FES has been shown to offer several benefits for individuals with SCI. First, it can help prevent muscle atrophy and maintain muscle mass by providing regular stimulation to the paralyzed muscles [46]. This can mitigate the secondary effects of SCI such as muscle weakness and contractures, thus preserving overall muscle function. Additionally, FES can facilitate task-specific training by enabling individuals to perform functional movements that would otherwise be impossible due to paralysis. For example, FES-assisted cycling can improve cardiovascular fitness, lower extremity strength, and range of motion in individuals with SCI. Moreover, FES can enhance neuroplasticity and promote reorganization of the central nervous system by providing sensory feedback and activating residual neural pathways [47]. This can lead to improvements in motor control, coordination, and balance over time. Regarding motor recovery, FES has shown promising results in enabling individuals with SCI to regain voluntary muscle control and functional mobility. Studies have demonstrated that FES can improve muscle strength, endurance, and coordination, allowing individuals to perform activities of daily living more independently [48]. Furthermore, FES-assisted gait training has been shown to enhance walking ability and reduce the reliance on assistive devices in some individuals with incomplete SCI [49]. Overall, FES holds significant potential as a rehabilitation tool for promoting motor recovery and improving the quality of life for individuals living with SCI. Ongoing research continues to explore novel applications of FES and optimize its efficacy in SCI rehabilitation.

Over the past few decades, numerous clinical trials have investigated the effectiveness of FES in various medical conditions, including SCI, with their findings summarized in recent reviews. By applying an additional filter focusing on the last decade, we can highlight specific clinical trials where FES was applied and yielded outcomes related to motor recovery.

In a clinical trial led by Triolo et al., involving six individuals, outcomes demonstrated that stabilizing the pelvis and trunk through the application of FES to specific pelvic muscles reduced effort and positively impacted manual wheelchair propulsion [50]. Restoration of seated posture during a forward fall was described in five individuals by Murphy et al. when FES was applied [51]. In a clinical trial involving 25 subjects conducted by Sadowsky et al., the combination of FES with cycling elicited a response in the American Spinal Injury motor score [52]. In a clinical trial by Kapadia et al., FES combined with assisted walking demonstrated improvements in both the Spinal Cord Independence Measure and the Functional Independence Measure in 16 patients [53]. Osuagwu et al. described a positive effect on neurological and, to some extent, on functional recovery in seven patients when combining FES with brain-computer interface (BCI) [54]. Synergic and functional hand movements were described by Coste et al. when specific nerves were stimulated in two subjects [55]. Memberg et al. detected the restoration of arm and hand functions for ADLs in two patients [56]. Furthermore, Duffell et al. featured improved International Standards for Neurological Classification of Spinal Cord Injury (ISNC-SCI) motor scores in chronic patients in a clinical trial using a combination of FES-cycling and biofeedback [57]. Moreover, support for front crawl swimming was described when FES and tSCS were applied in a clinical trial by Wiesener et al. [58]. In another trial when FES and BCI combination was applied, intuitive control over reaching and grasping movements was provided as an outcome by Ajiboye et al. [59]. Finally, isolated, compound, functional, and strong movements were observed when neural implants were placed in specific nerves in an eight-subject clinical trial by Tigra et al. [60].

Table 3 summarizes the FES studies incorporated in this analysis, presenting the demographic characteristics, injury type/level, stimulator type and location, and the primary outcomes of each study.

**Table 3:** Overview of functional electrical stimulation studies. FES = functional electrical stimulation; AIS = American Spinal Injury Association Impairment Scale; NS = not specified; M = male; F = female; C = cervical; T = thoracic; L = lumbar; S = sacral; BCI = brain-computer interface; ES = erector spinae; GM = gluteus maximus; QL = quadratus lumborum; SM = semimembranosus; AM = posterior portion of adductor magnus; GMed = gluteus medius; SCIM = Spinal Cord Independence Measure; FIM - Functional Independence Measure; ADLs = activities of daily living; ISNCSCI = International Standards for Neurological Classification of Spinal Cord Injury

Study	Design	Subjects	Sex	Time	Intervention	Level - AIS	Stimulator type	Location of stimuli	Outcome
Triolo et al. (2013) [50]	Clinical trial	6	4 M, 2 F	Chronic	FES	T6 AIS B, T6 AIS A, C7 AIS B, T6 AIS B, C6 AIS A, T10 AIS A	IRS-8 IST-16	Muscles: ES, GM, QL, SM, AM	Stabilized the pelvis and trunk, reduced effort, and impacted manual wheelchair propulsion for all subjects
Murphy et al. (2014) [51]	Clinical trial	5	2 M, 3 F	Chronic	FES	C7 AIS C, C7 AIS B, T5 AIS B, T6 AIS A, T10 AIS A	IRS-8 IST-16	Muscles: AM, ES, GMed, QL, SM	Restored seated posture during a forward fall in all subjects
Sadowsky et al. (2013) [52]	Randomized clinical trial	25	22 M, 3 F	chronic	FES - cycling	C1-T1 (13), T2-L5 (12), AIS A (17), AIS B (5 ), AIS C (3)	NS	Quadriceps, gluteal, and hamstring	AIS motor score response in 20 subjects
Kapadia et al. (2014) [53]	Randomized controlled trial	16	12 M, 4 F	Chronic	FES - assisted walking	C2-T12 AIS C-D	Compex SA, Switzerland	Bilateral quadriceps, hamstrings, dorsiflexors, and plantarflexors	SCIM and FIM improved in each subject
Osuagwu et al. (2016) [54]	Randomized clinical trial	7	M	subacute	FES + BCI	C6 AIS C, C4 AIS B, C6 AIS B, C5 AIS C, C6 AIS C, C5 AIS B, C6 AIS C	Guger Technologies, Austria	NS	Positive effect on neurological and to some extent on functional recovery in all subjects
Azevedo Coste et al. (2022) [55]	Clinical trial	2	NS	Chronic	FES	C4 AIS A (both)	CorTeC GmbH, Freiburg, Germany	Median nerve, radial nerve	Synergic and functional hand movements reported for both subjects
Memberg et al. (2014) [56]	Case study	2	1 M, 1 F	Chronic	FES	Brown–Séquard injury at the C1-C2, C3 AIS A	IST-12	Shoulder and arm	Restoring arm and hand functions for ADLs in both subjects
Duffell et al. (2019) [57]	Clinical trial	11	10 M, 1 F	Five subacute, six chronic	FES cycling + biofeedback	C1-T12 AIS C-D	Odstock Medical Ltd., UK	Quadriceps, hamstrings, gluteals	Improved ISNCSCI motor score in six (chronic) subjects
Wiesener et al. (2020) [58]	Clinical trial	2	NS	Chronic	FES + tSCS	T5 AIS A, T6 AIS A	RehaMove3, Hasomed GmbH, Germany	Back	Supported front crawl swimming in both subjects
Ajiboye et al. (2017) [59]	Clinical trial	1	M	Chronic	FES + BCI	C4 AIS A	Blackrock Microsystems + Synapse Biomedical	Precentral gyrus	Intuitive control over reaching and grasping movements
Tigra et al. (2020) [60]	Clinical trial	8	NS	Chronic	Neural implants	C5 AIS A	Cortec GmbH, Freiburg, Germany	Radial nerve, median nerve	Isolated, compound, functional, and strong movements reported in all subjects

Limitations

However, this study has a limitation regarding the number of clinical trials conducted and the number of participants included, which may affect the reliability of the outcomes. Therefore, despite the encouraging results, ongoing research efforts are needed to optimize stimulation parameters, refine treatment protocols, and elucidate the underlying mechanisms of action. Additionally, large-scale clinical trials and long-term follow-up studies are warranted to validate the efficacy and safety of these interventions across diverse patient populations and injury severities.

## Conclusions

In conclusion, electrical stimulation techniques, including eSCS, tSCS, and FES, have emerged as promising modalities for promoting motor recovery in individuals with SCI. These techniques harness the inherent plasticity of the nervous system to facilitate functional improvements below the level of injury. eSCS targets specific motor circuits within the spinal cord, modulating neural activity and promoting voluntary movement. Similarly, tSCS delivers non-invasive electrical impulses to the spinal cord through surface electrodes, enhancing spinal excitability and facilitating motor function. Meanwhile, FES directly stimulates nerves and muscles, enabling task-specific movements and promoting muscle strength and coordination.

The reviewed clinical trials have demonstrated the diverse applications and efficacy of electrical stimulation in motor recovery. From facilitating standing and walking to enhancing upper extremity functionality and trunk stability, these interventions have shown promising outcomes in improving functional abilities and quality of life for individuals with SCI. Moreover, the studies highlight the potential of combining electrical stimulation techniques with adjunct therapies, such as pharmacological agents or exoskeletons, to further enhance motor recovery outcomes.

Ultimately, the integration of electrical stimulation techniques into comprehensive rehabilitation programs holds great promise for maximizing motor recovery and improving outcomes for individuals living with SCI. Continued collaboration between clinicians, researchers, and engineers will be essential in advancing the field and translating these findings into clinical practice.
